# Reopening of dental clinics during SARS-CoV-2 pandemic: an evidence-based review of literature for clinical interventions

**DOI:** 10.1186/s40902-020-00268-1

**Published:** 2020-08-03

**Authors:** Seied Omid Keyhan, Hamid Reza Fallahi, Amin Motamedi, Vahid Khoshkam, Paymon Mehryar, Omid Moghaddas, Behzad Cheshmi, Parsa Firoozi, Parisa Yousefi, Behzad Houshmand

**Affiliations:** 1 CMFRC, National Advance Center for Craniomaxillofacial Reconstruction, Tehran, Iran; 2 Craniomaxillofacial Research Center, Tehran University of Medical Sciences, Tehran, Iran; 3 Dental Research Center, Research Institute of Dental Sciences, Shahid Beheshti University of Medical Sciences, Tehran, Iran; 4 Private Practice, Kerman, Iran; 5 Private Practice, El Paso, TX USA; 6 Private Practice, Austin, TX USA; 7 Department of Periodontology, Islamic Azad University, Tehran, Iran; 8 Faculty of Dentistry, Boroujerd Islamic Azad University, Boroujerd, P.O 6915136111 Iran; 9 Faculty of Dentistry, Department of Oral and Maxillofacial Surgery, School of Dentistry, Zanjan University of Medical Sciences, Zanjan, Iran; 10 Resident of prosthodontics, Isfahan University of Medical Sciences, College of Dentistry, Isfahan, Iran; 11 School of Dentistry, Shahid Beheshti University of Medical Sciences, Tehran, Iran

**Keywords:** SARS-CoV-2, COVID-19, Dental clinics, Dentistry

## Abstract

**Background:**

Severe acute respiratory syndrome coronavirus 2 (SARS-CoV-2) causes serious acute respiratory diseases including pneumonia and bronchitis with approximately 2.3% fatality occurrence.

**Main body:**

This study argues the main concepts that need to be considered for the gradual reopening of dental offices include treatment planning approaches, fundamental elements needed to prevent transmission of SARS-CoV-2 virus in dental healthcare settings, personal protection equipment (PPE) for dental health care providers, environmental measures, adjunctive measures, and rapid point of care tests in dental offices.

**Conclusion:**

This article seeks to provide an overview of existing scientific evidence to suggest a guideline for reopening dental offices.

## Background and history

Coronaviruses (CoVs) are the largest group of known positive-sense RNA viruses with a variety of hosts in nature [1]. In the beginning, coronaviruses were thought to cause only enzootic infections in several animals, including a community of birds and mammals. However, current studies have shown that these viruses are infectious in humans [2, 3]. Seven major coronaviruses (CoVs) were recognized by 2020, including SARS-CoV-2. Within these 7 viruses, three of them (SARS-CoV, MERS-CoV, SARS-CoV-2) lead to serious respiratory syndromes with considerable death rates [4–6]. In 2002, SARS-CoV extended over five continents with a 10% death rate, and in 2012, MERS-CoV emerged with a 35% fatality rate in the Arabian Peninsula [7]. A novel coronavirus (SARS-CoV-2) causes serious acute respiratory diseases including pneumonia and bronchitis with approximately 2.3% fatality occurrence [8, 9]. The most prevalent signs and symptoms are cough (76%), fever (98%), myalgia or fatigue (44%), and dyspnea (55%). The SARS-CoV-2 incubation period has been reported to be 1–14 days, and asymptomatic individuals may also involve in the spread of this virus [10–13]. Due to the significant human-to-human route of contamination of these coronaviruses, dentists according to their close contact with patients are at danger of SARS-CoV-2 in dental procedures [5]. Even though all routine dental treatment in countries with SARS-CoV-2 infection has been postponed during the pandemic era, the need for emergency care provided by teams with sufficient personal protective equipment takes priority [14].

## Treatment planning approaches

As a general principle, all non-emergent dental care for all individuals should be deferred during the pandemic crisis [15]. However, since the social demand for emergency care services even during this situation will be crucial [16], in situations that are considered an emergency according to the therapist’s judgment, the patient should be called to the office for the most possible conservative treatment [17, 18]. Dentists must be available to their patients for emergency care. Therefore, during pandemics, considering the infection control principles, our approach to treatments can be classified into four main categories:

1. Treatments that can be accomplished using their standard conventional approach.
2. Treatments that can be accomplished using a modified approach.
3. Interim treatments that are performed only to eliminate severe pain or potentially serious patient’s life- or health-threatening conditions so that definitive treatment can be accomplished later.
4. Treatments that should be avoided; instead, available alternative treatment options should be applied.

### Recommendations for clinical interventions

**Fig. 1:**
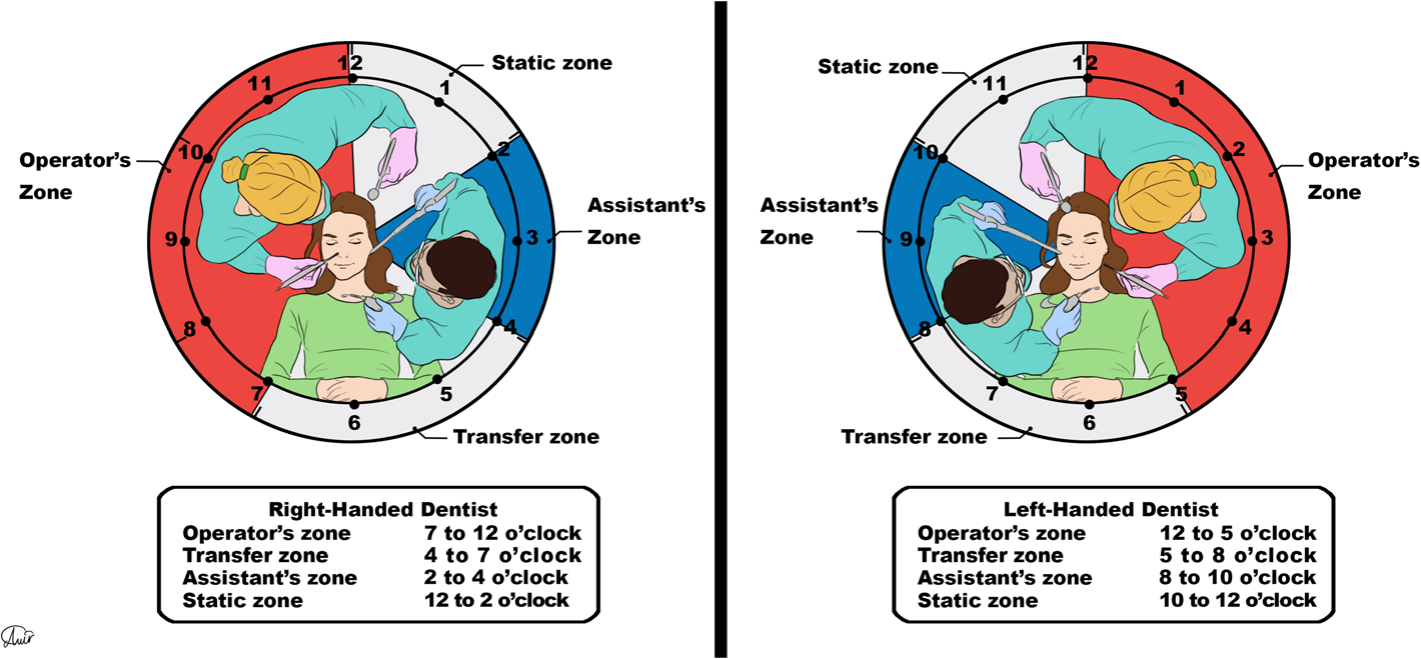
Four-handed dentistry. Zones of activity for right-handed and left-handed dentists

- If basic personal protection equipment (PPE), such as facemask and gloves, are not available, regardless of the urgency of the condition, any treatment cannot be proceeding.
- For patients who are incapable of mouthrinsing (such as children), the application of a rubber dam for aerosol-generating procedures is recommended. In addition, cotton rolls soaking is a suitable substitute for the pre-procedural rinsing.
- Avoid or minimize procedures that may induce coughing, such as taking intraoral x-rays. Instead, extra oral radiographs, such as panoramic and CBCT, are proper alternatives because they will not induce coughing.
- Aerosol-generating treatments are strongly recommended to be avoided.
- Manual instruments are an appropriate alternative to minimize the generation of aerosols.
- Sometimes, for patients with a serious emergency condition, it may be an inevitable option to extract highly infectious teeth with a questionable prognosis that under normal circumstances could have had their prognosis improved by being treated.
- Also, when definitive treatment is not possible due to infection control considerations, pulpotomy using MTA can be a good alternative for root canal therapy (RCT) for the management of symptomatic mature permanent teeth.
- To minimize the generation of droplets and aerosols in cases that aerosol-generating treatments are irreplaceable, four-handed operation (Fig. 1), high evacuation ejector, and rubber dam can be beneficial.
- Spatter-generating treatments are advised to be scheduled as the last appointments of the shift.
- As a serious source of cross-infection, dental health care providers should know that backflow may occur during the use of a saliva ejector.
- To minimize the need for recall appointments, the use of resorbable sutures is recommended.
- Consider avoiding the application of instruments that are not easily disinfected (Table 1) [19–29].

**Table 1:** Factors related to aerosol contamination

Device, instruments, measures	Description
**Ultrasonic and sonic scalers**	• They generate the highest rate of aerosols in a 6–12-in diameter from the operator. • The application of high volume evacuators reduces airborne contamination by 95%.
**Air polishing**	• Airborne contamination of air polishing is almost equal to ultrasonic scalers. • The application of high-volume evacuator and/or aerosol reduction device reduces airborne contaminations up to 95%.
**Air-water syringe**	• Airborne contamination of air-water syringes is almost equal to ultrasonic scalers. • However, the application of high-volume evacuators can reduce airborne contamination by nearly 99%.
**High speed air turbine handpiece**	• An air-driven handpiece is powered by compressed air to spin the air-driven turbine. • Air-driven handpieces reach speeds of up to 400,000 rpm in a variety of torques. • The water flow speeds for turbines with one, two, and three coolant apertures are 42.38, 34.31, and 30.44 mL/min, respectively, or about 1.0 ml/s. • High-speed dental handpieces without anti-retraction valves aspirate the debris and fluids and contaminate the air and water systems of the unit which may lead to cross-infection.
**Slow speed handpieces**	• The speed of their inbuilt motor can reach up to 80,000 rpm. • The average pressure for air and external water in these handpieces are about 0.25–0.3 (Mpa) and 198 (Kpa), respectively. • The average water flow in these handpieces is about 90–110 (min/ml).
**Anti-retraction high-speed dental handpiece**	• These handpieces reduce the microbial backflow into the tubes of the handpieces and dental units.
**Electric motor handpieces**	• Self-contained internal gearings in an electric motor handpiece enable it to function at a stable torque and speeds up to 200,000 rpm.
**Tooth preparation with air abrasion**	• Extensive microbial contamination with abrasive particles has been demonstrated.
**Aerosol reduction device**	• Aerosol reduction devices such as Jet Shield (DENTSPLY SIRONA INC., USA) can reduce the contamination up to 97% during air polishing.
**Rubber dam**	• Rubber dams minimize the formation of the blood- and saliva-contaminated aerosols. • The application of rubber dam reduces airborne particles in ~ 1-meter diameter of the source of particle production by 70%.
**High-volume evacuator**	• High-volume evacuators can efficiently reduce the number of microorganisms, blood, and material released into the air. • Since a small-bore saliva ejector is not an adequate substitute, when a four-handed operation is not an option, utilization of a high-volume evacuator attached to the instrument is necessary. • The use of these types of evacuators when the utilization of a rubber dam is impractical can be highly beneficial. •Researches indicated that the application of high-velocity evacuators with air polishers can reduce CFUs about 94.8%.
**Ultraviolet radiation**	• Ultra-violet radiation can be considered as a highly fungicidal, viricidal, and bactericidal agent via damaging DNA and denaturation of proteins. • The International Ultraviolet Association (IUVA) stated that UV disinfection can reduce the transmission of the SARS-CoV-2 in air, water, and on surfaces.
**Patient and dentist position**	• The patient in the supine position enables the dental team to stay away from the patient’s breathing way.
**Ventilation and air-conditioning system**	• To reduce environmental contamination and prevent contaminated air circulation
**Microbiological control of unit water system**	• Periodic disinfection of the unit water system via application of chemicals and distilled water.
**Preprocedural mouth-rinse**	• The efficiency of chlorhexidine on SARS-CoV-2 has not yet been studied. However, the use of 1% hydrogen peroxide or 0.2% povidone-iodine is recommended. • Prophylactic administration of mouthwash reduces the microbial load in the oral cavity.
**Manual instruments**	• Manual instruments are recommended to minimize the aerosol generation.

## Fundamental elements needed to prevent transmission of SARS-CoV-2 virus in dental healthcare settings

### Administrative measures

Administrative measures are all infrastructures to implement, guide, support, and monitor adherence to standard and Transmission-Based Precautions. A key administrative measure is provision of necessary and sufficient fiscal and human resources for maintaining infection control (IC) programs. Specific components include adequate staff, inclusion of infection control practices (ICP) in dental facility construction and design decisions, adequate supplies, equipment, and compliance monitoring [30].

### Infection control practice landscape in dental settings

Studies show that in general, knowledge and attitudes regarding infection control are good; however, the compliance and practice levels regarding the same are low [31–36]. Findings indicate that lack of compliance with ICP is multifactorial, and compliance with recommended IC guidelines is challenging, and the results of some studies indicate that compliance is achievable, even in medium and large group practices [32, 37–39].

### Organizational and individual factors that affect ICP compliance

A review of the literature concluded that variations in organizational factors (e.g., safety climate, policies and procedures, education and training, adequate resourcing, innovation culture, staff education, and adequate highly trained and experienced staff) and individual factors (e.g., knowledge, perceptions of risk, and past experience) were determinants of adherence to ICP for protection against SARS and other respiratory pathogens [40–43].

### Education of dental health care providers (DHCP)

A study on Hong Kong hospital workers demonstrated that the likelihood of SARS infection was strongly associated with having less than 2 h of infection control (IC) training and not understanding infection control procedures [44]. It is important to realize that IC education and training goal are not a simple memorization of protocols. Further attempts to fill the gap between knowledge and practice change should be made [43]. Implementing problem-based learning, evidence-based practice methods, practical demonstration and participation actions, incorporating individual experience, and hands-on training is associated with decreased healthcare-associated infections (HAI) and hand hygiene compliance [45–47]. Strong recurrent HCP education and training with the aim to reduce specific types of infections is effective for guideline implementation [47].

## Personal protection equipment (PPE) for DHCP

**Fig. 2:**
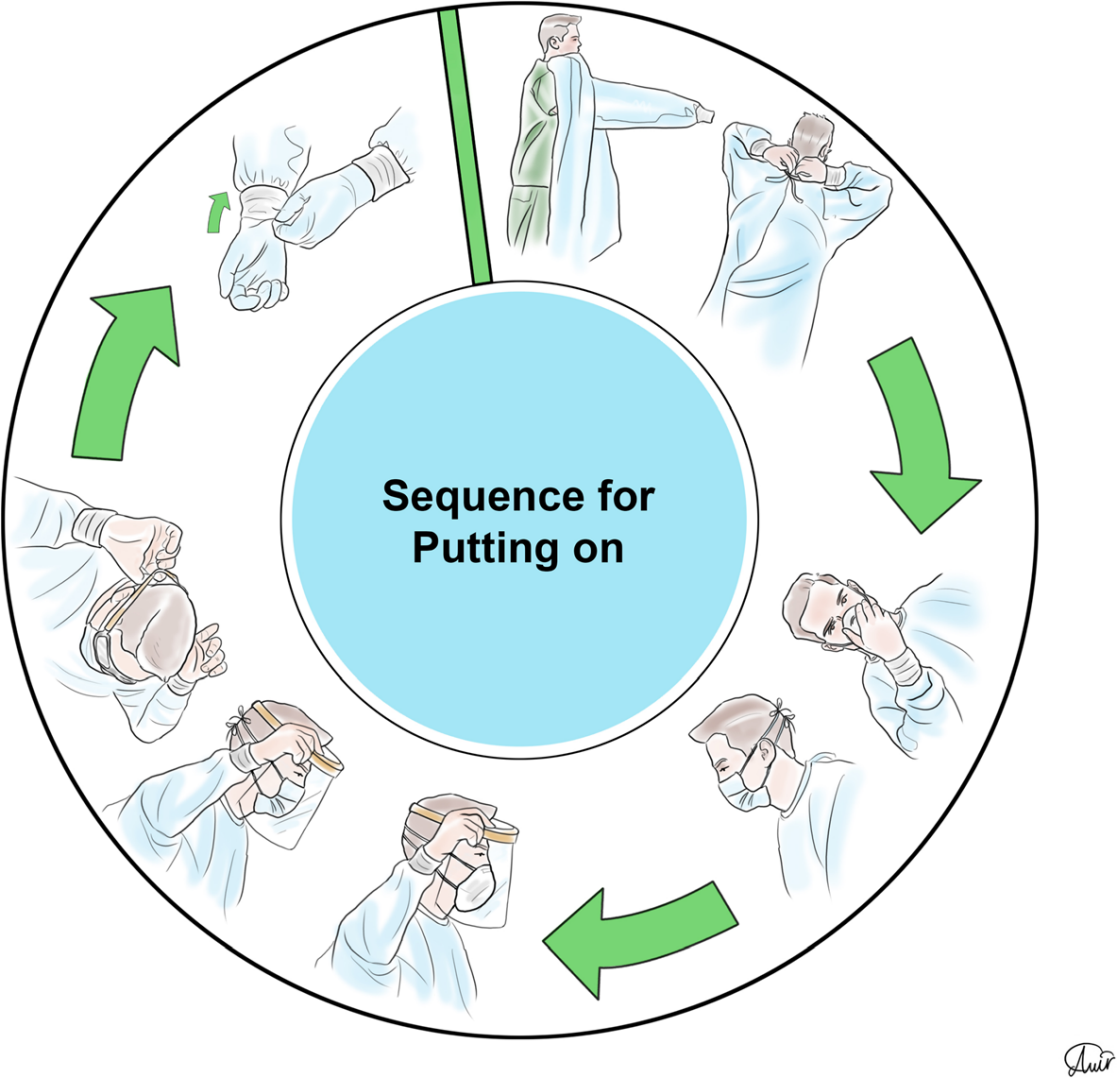
Personal protection equipment donning order

**Fig. 3:**
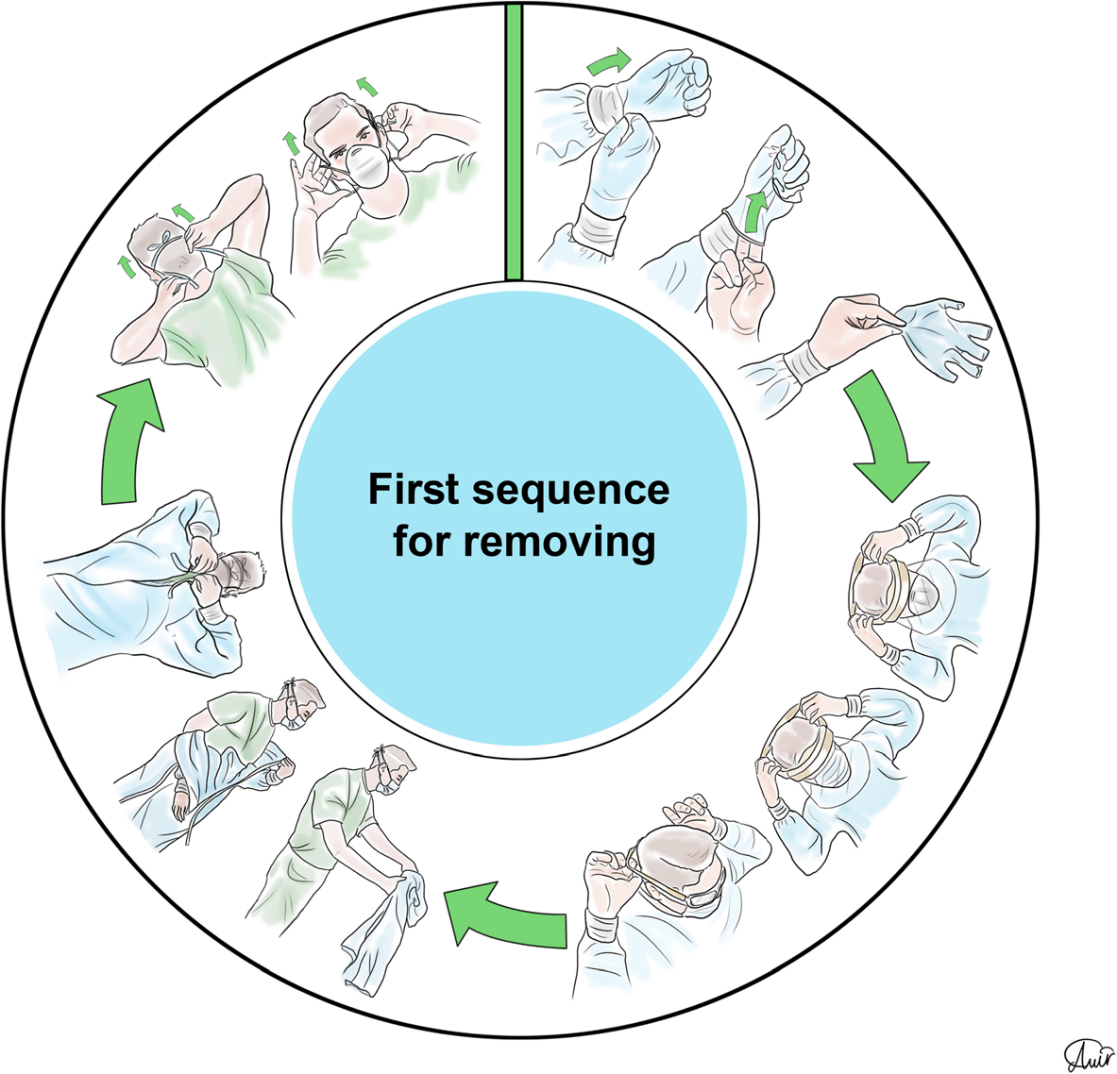
Personal protection equipment doffing first order

**Fig. 4:**
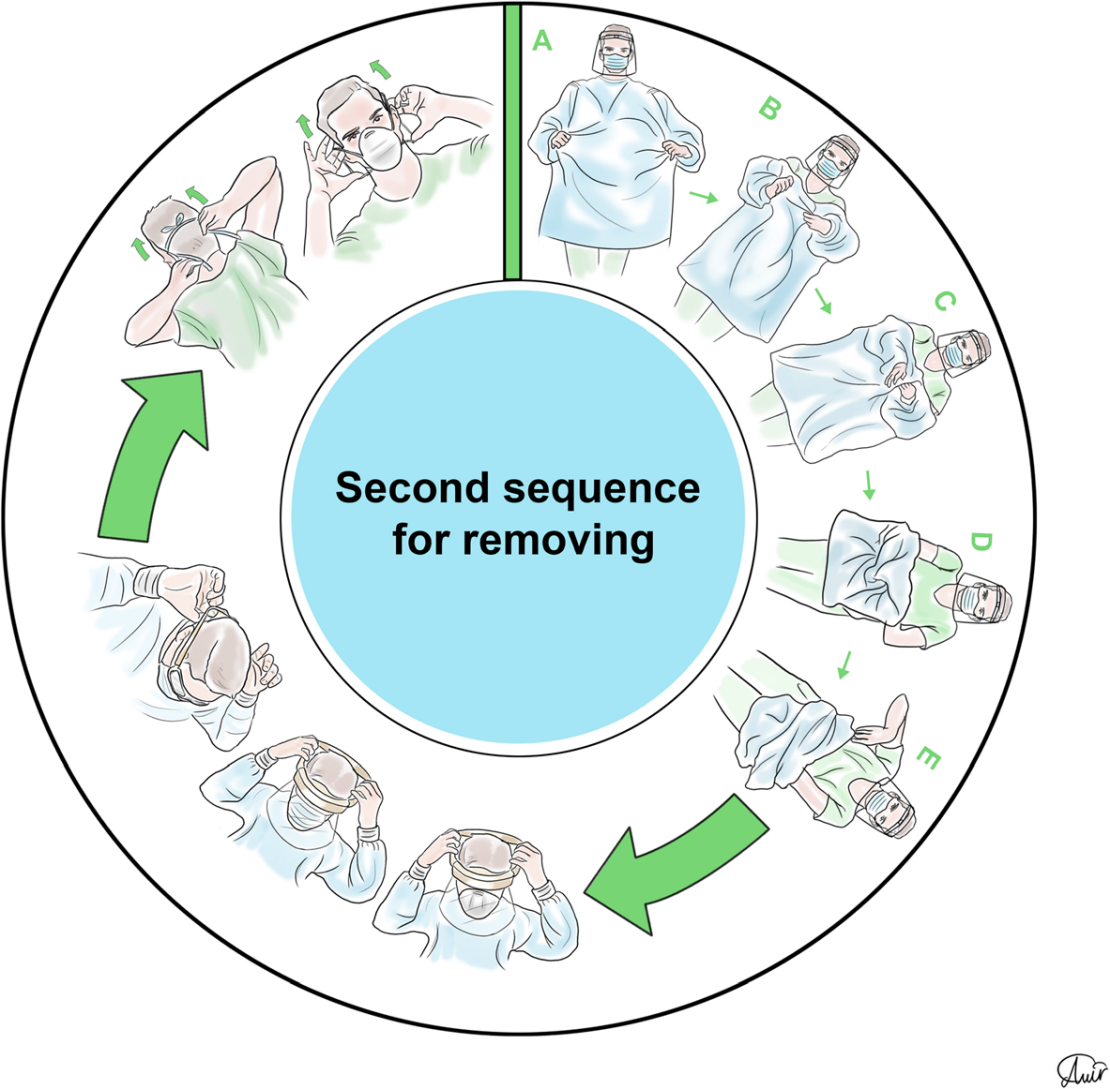
Personal protection equipment doffing second order

**Fig. 5:**
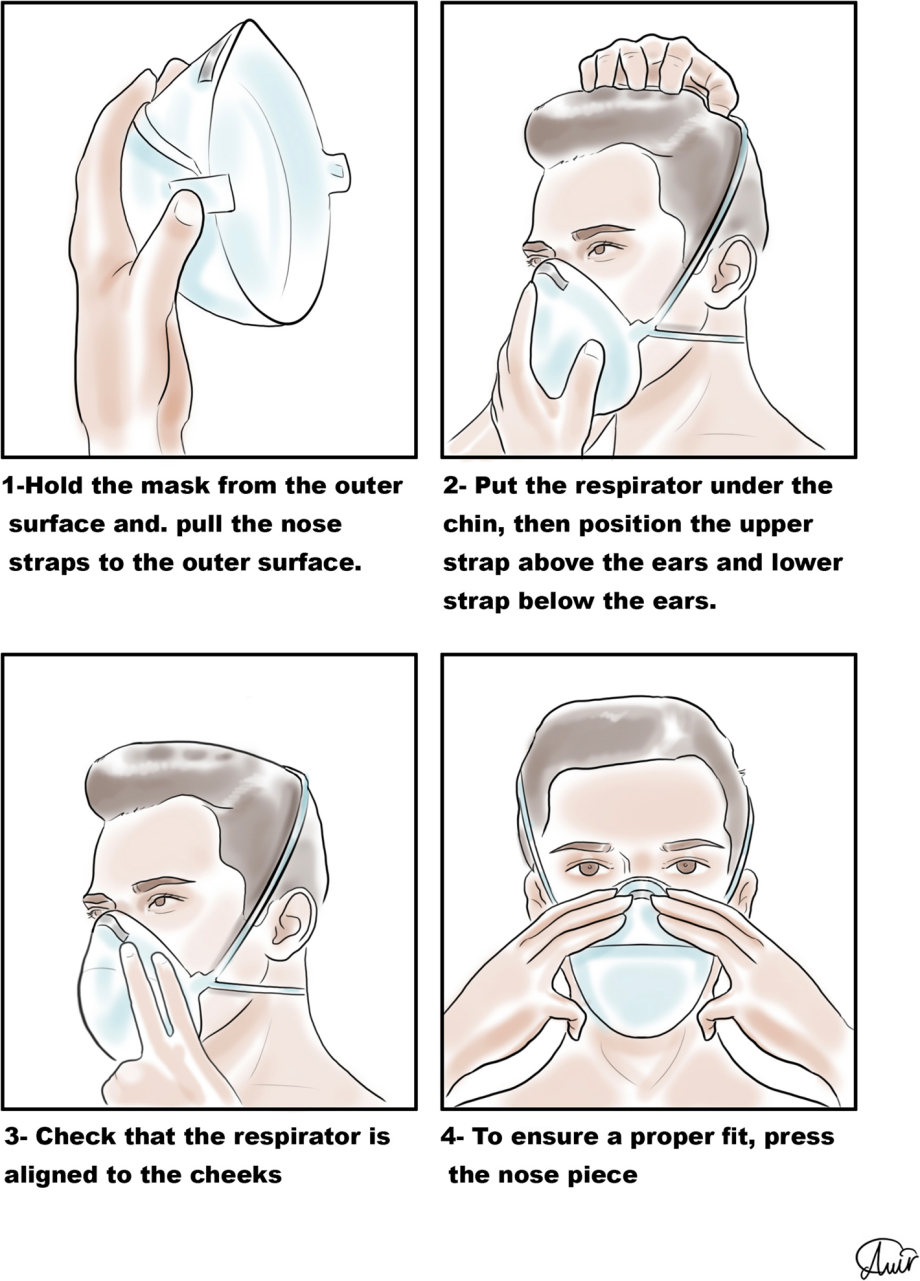
Correct way of putting on and removing a respirator

Personal protection equipment (PPE) reduces the risk of contamination, and healthcare workers should take this issue seriously [48]. For SARS-CoV-2, recommendations for PPE are masks, respirators, gloves, goggles or face shields, and long gowns [49, 50]. More body coverage leads to better protection. Donning and doffing of PPE should be easy [51] since the complexity of use leads to an increased risk of self-contamination especially during doffing [52]. The correct sequence of donning and doffing is depicted in Figs. 2, 3, 4, and 5.

### Masks and respirators

Waterproof surgical masks prevent the spread of respiratory droplets in the environment and protect staff against both infected droplets and contact contamination. Also, they reduce the risk of SARS-CoV-2 contamination by at least 80% [2]. Filtering facepiece respirators (FFRs) including N95 respirators is known as effective and protective equipment that their filtration has been achieved via a network of polypropylene microfibers and electrostatic charges [53]. A meta-analysis revealed that there is no statistically significant difference between surgical masks and facepiece respirators such as FFPs and N95 in terms of protection against airborne viral infections (RR = 0.89, *p* > 0.05) [54]. Powered air-purifying respirator (PAPR) is also recommended for protection against SARS-CoV-2 [55]. However, due to the electronic nature of this device and the possibility of damage to the electronic parts of it, it is recommended to use it simultaneously with a filtering facepiece respirator [56]. Reusable elastomeric respirators are not commonly used in health care settings and are used widely in the industry and are available in full-face, half-face, and quarter-face models [57]. Comparisons between different masks and respirators are shown in Table 2 [57].

**Table 2:** A brief comparison between masks and respirators

Mask type	Standard	Filtration effectiveness			Re-usability
**Single-use medical masks**	China: YY/T0969	3.0 microns: > 95% 0.1 microns: not effective			No
**Surgical masks**	China : YY 0469	3.0 microns: > 95% 0.1 microns: > 30%			No
**Surgical masks**	USA: ASTM F2100	Level 1	Level 2, 3		No
		3.0 microns: > 95% 0.1 microns: > 95%	3.0 microns: > 95% 0.1 microns: > 95%		
**Surgical masks**	Europe: EN 14683	Type 1	Type 2,3		No
		3.0 microns: > 95% 0.1 microns: > 95%	3.0 microns: > 95% 0.1 microns: > 95%		
**Respirator masks**	USA: NIOSH 42 CFR 84	N95	N99	N100	Yes (under especial conditions)
		0.3 microns: > 95%	0.3 microns: > 99%	0.3 microns: > 99.97%	
**Respirator masks**	Europe: EN 149: 2001	FFP1	FFP2	FFP3	Yes (under especial conditions)
		0.3 microns : >80%	0.3 microns : >94%	0.3 microns : >99%	
**Elastomeric respirators**	USA: NIOSH 42 CFR 84	10 to 50 APF			Yes
**PAPR**	USA: NIOSH 42 CFR 84	1000 APF			Yes

***PAPR*** powered air-purifying respirator*.* ***APF*** assigned protection factor

Due to the SARS-CoV-2 pandemic and the reduction in access to face masks and respirators such as the N95, the CDC recommends methods for extended use and reuse of them [58]. For extended use, the CDC recommends using an N95 respirator for up to 8 h; however, it is recommended to follow the manufacturer’s instructions. Based on CDC, it should be noted that FFRs can be reused up to 5 times via the following strategies:

1. Mask rotation: In this technique, the masks must be numbered and used in turn. The minimum time for not using a used mask should be at least 72 h, as the SARS-CoV-2 loses its viability. However, if a mask is damaged or used in the aerosol-generating process, it should be discarded.
2. Reprocessing/decontamination: Hydrogen peroxide vaporization can be used on N95 models that do not contain cellulose, such as the 1860 model. Also, methods such as proper UV treatment of N95 masks, moist heat (heating at 60–70 °C and 80–85% relative humidity), and dry heating of the mask at 70 °C for 30 min can be used for decontamination; however, dry and moist heat is not currently recommended for SARS-CoV-2.

### Gowns

Different qualities have been reported for gowns [59]. Most models of isolation gowns often leave the neck exposed, which can be a route of contamination [60]. The most protection is assigned to coveralls followed by long gowns, gowns, and aprons, respectively [51]. According to the studies, modified gowns with attached gloves, cover the wrist area, and gowns that fit tightly at the neck area reduce the risk of contamination in the best way [51]. It is also recommended that the gowns be removed simultaneously with the gloves [51].

### Gloves

**Fig. 6:**
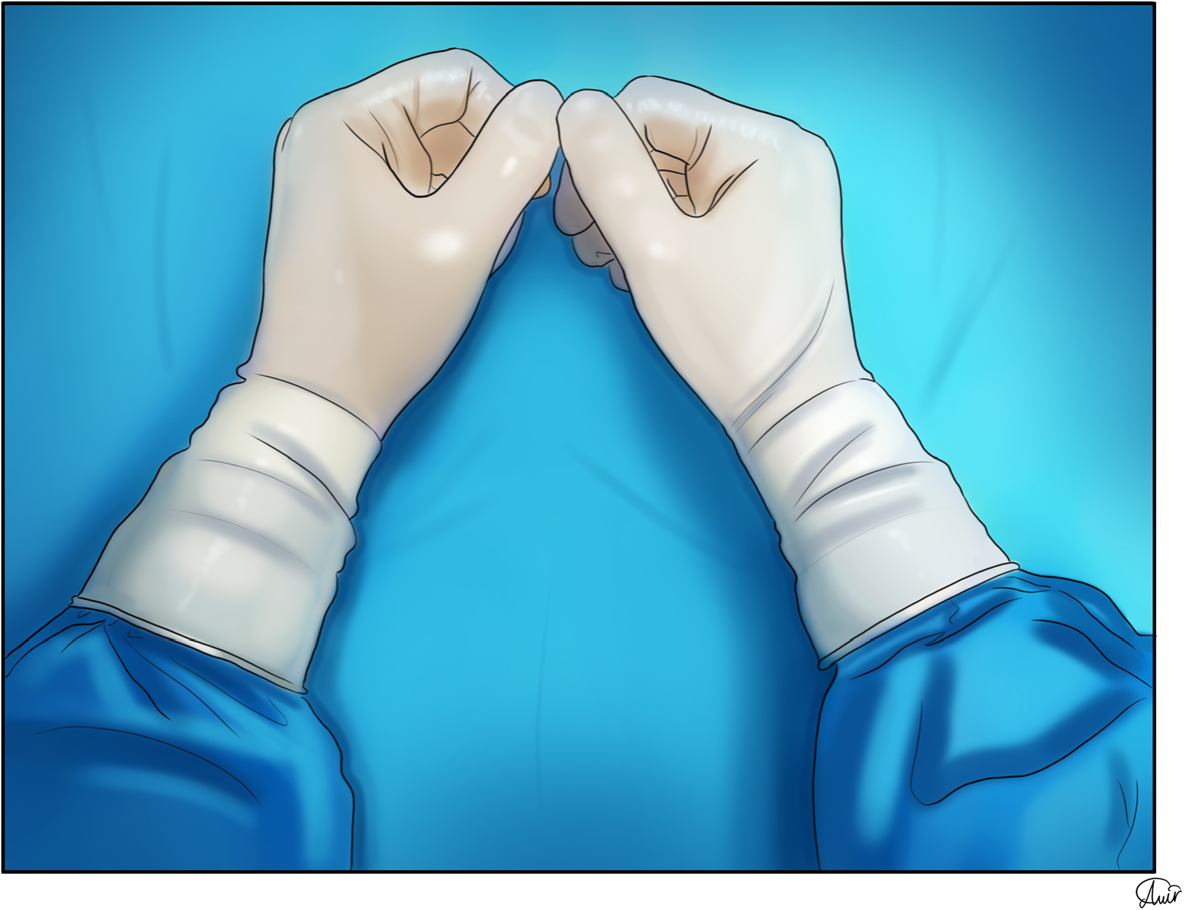
Arm-length surgical gloves that completely cover the wrist area

Adding tabs to the gloves for taking them off from the hands reduces the risk of contamination [51]. Studies showed that the risk of contamination using double or triple gloves is less than single glove. Also, donning three layers of gloves due to the complex doffing process is not suggested due to more risk of self-contamination [61, 62]. Cleaning of gloves with hypochlorite or quaternary ammonium except alcohol-based hand rubs may decrease hand contamination [51]. Dentists should use arm-length surgical gloves (Fig. 6) [63].

### Eye protectors

**Fig. 7:**
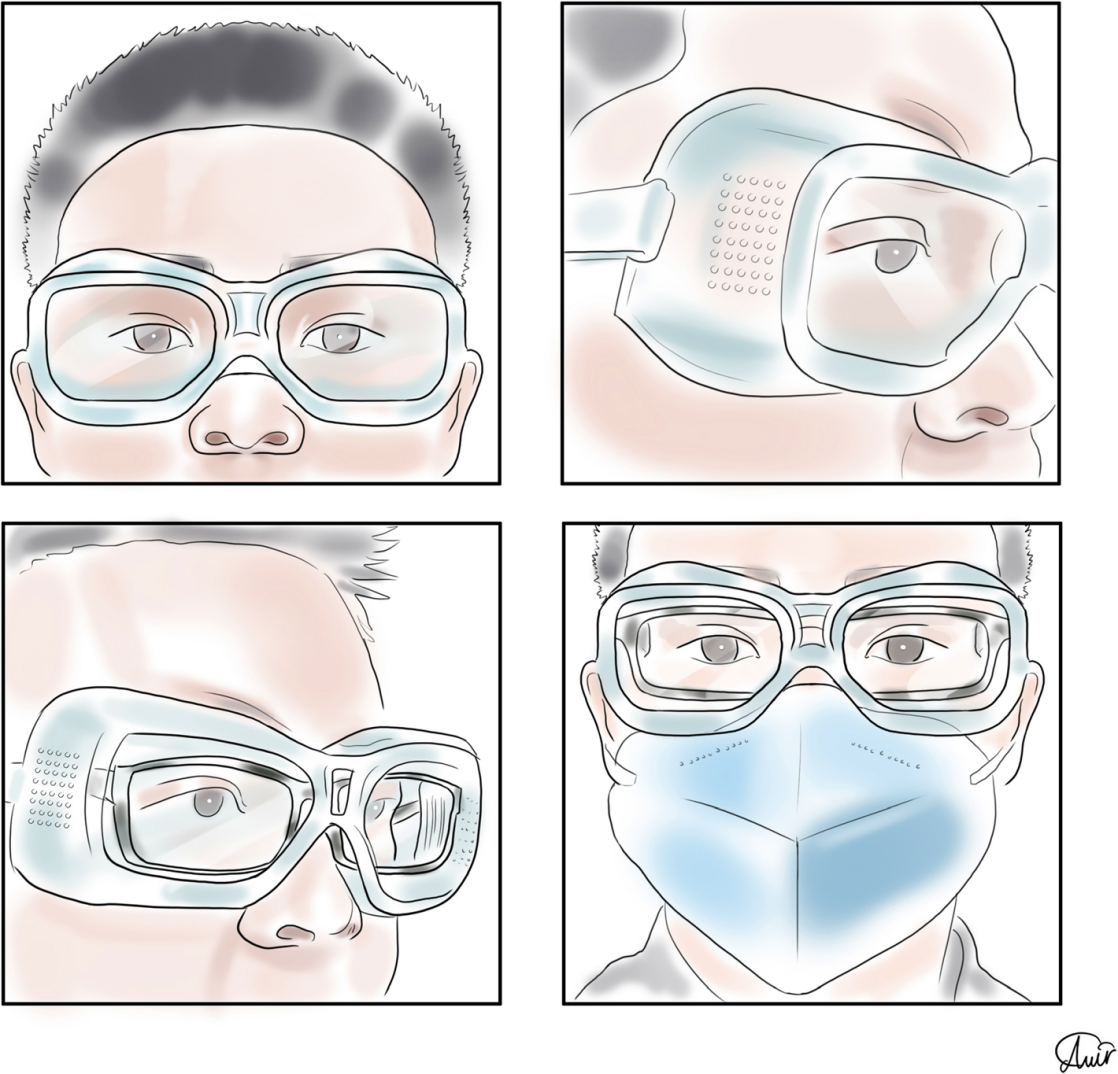
A proper goggles provide a complete eye seal

Lindsley et al. used breathing and coughing simulators to determine the efficacy of face shields in reducing contamination. They proved that face shields are effective in reducing the exposure to large infectious particles, but smaller particles are able to remain airborne and flow around a face shield to be inhaled [64]. Face shields are more bulky than goggles and protect the entire face [64]. Figure 7 shows a standard eye protector providing full eye seal.

## Hand hygiene

**Fig. 8:**
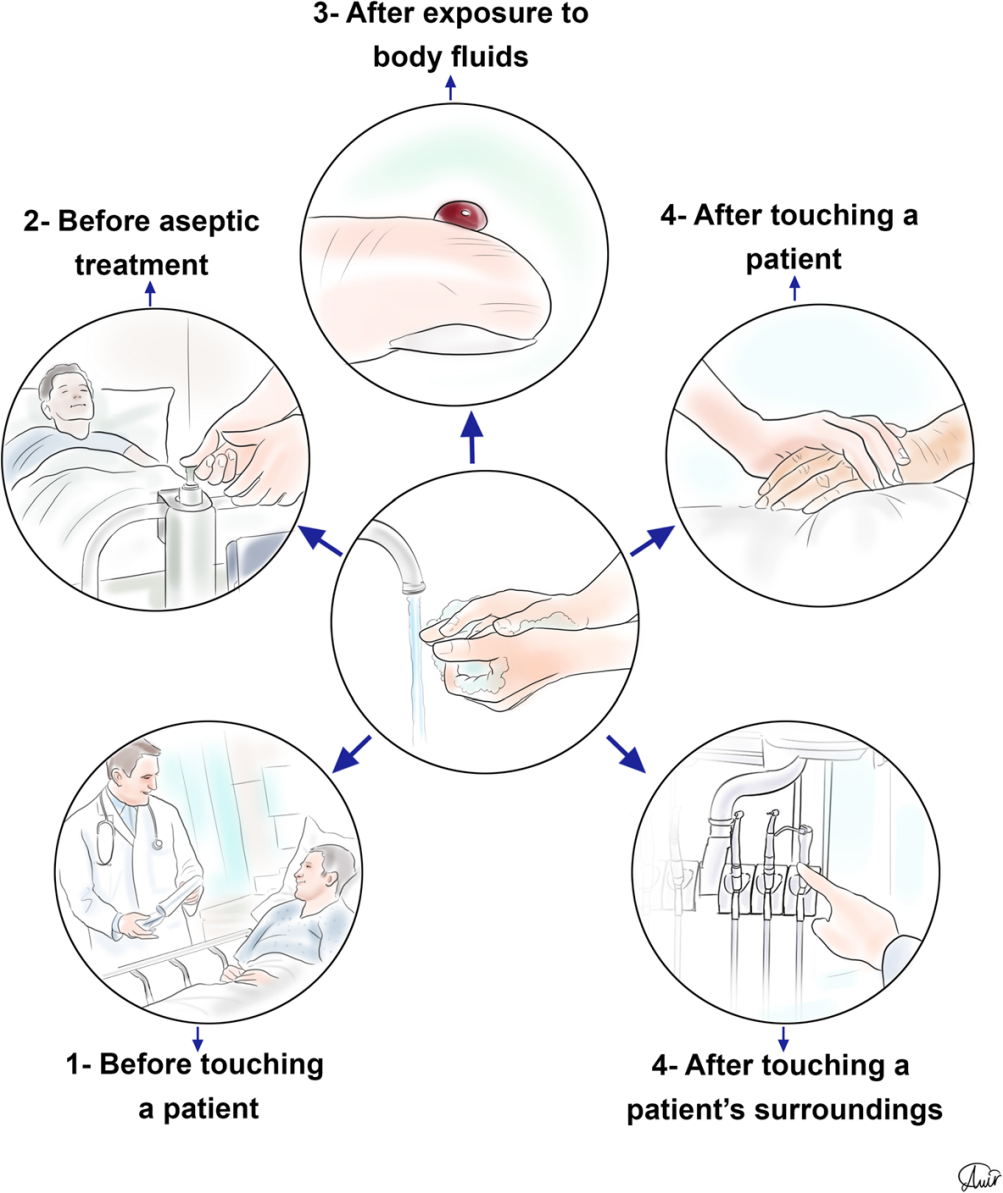
Five main times that hand hygiene should be considered seriously

It has been shown that hand hygiene does not provide an adequate defensive response to viruses without the use of face masks [65]. Ethanol is widely used in the world for hand rubbing in various forms including gels and foams [66]. Also, using alcohol-based disinfectants are promising substances to protect healthcare workers against SARS-CoV-2 [67]. The mechanism of alcohol-based sanitizers is denaturing proteins so that enveloped viruses including coronaviruses are removed by using these sanitizers [68]. Reports demonstrated that alcohol-based hand rubs could contain at least 60% ethanol to provide effective protection [69]. In 5 moments, healthcare workers should consider hand rubbing seriously: before touching a patient, before aseptic treatments, after exposure to body fluids, after touching a patient, and after touching the patients’ surroundings (Fig. 8) [70].

## Environmental measures

Cleaning and disinfecting non-critical surfaces in patient-care areas are part of standard precautions. In general, these procedures do not need to be changed for patients on transmission-based precautions and are appropriate for SARS-CoV-2 in healthcare settings, including those patient-care areas in which aerosol-generating procedures are performed. The cleaning and disinfection of all patient-care areas are important for frequently touched surfaces, especially those closest to the patient, that are most likely to be contaminated (e.g., dental chair, cabinets, doorknobs, desks, elevators, bathroom sinks, surfaces, and equipment in close proximity to the patient). The frequency or intensity of cleaning may need to change based on the patient’s level of hygiene and the degree of environmental contamination and for certain for infectious agents whose reservoir is the intestinal tract [71, 30, 72, 73]. A summary of the substances used for disinfecting and cleaning is presented in Table 3 [74–87].

**Table 3:** Methods of disinfecting non-critical surfaces in patient-care areas

Disinfecting non-critical surfaces in patient-care areas			
Vaporized hydrogen peroxide		Disinfectants	
Types	Virucidal efficacy	Hypochlorous acid (HOCl)	Other disinfectants
Non-condensing vaporized hydrogen peroxide (VHP) technology (Steris) and condensing search hydrogen peroxide vapour (HPV) technology (Bioquell)	Limited evidence is available for the virucidal activity of condensing HPV systems. Recently, several studies have demonstrated the in vitro activity of condensing HPV systems against individual viruses, including feline calicivirus (FCV), adenovirus, lactococcal bacteriophages6, and MS2 coliphage	**Virucidal efficacy**	Alkalis, oxidizing agents, alcohols, and aldehydes
		• Virucidal ability of solutions containing a high amount of HOCl is better than those containing HCl • Reduction of efficacy after spraying from a distance more than 30 cm • Minimum concentration should be more than 40 ppm for effective virucidal effect • The 100 and 200 ppm concentrated solutions inactivated more than 99.9% of AIV directly after spraying, while the 50 ppm concentration required at least 3 min of contact	

## Adjunctive measures

### Prophylactic medication for dental health care providers

Currently, there is no available and reliable evidence to support the prophylactic use of a medication(s) for dental health care providers, although a handful of trials in the world are being conducted to keep health care providers and vulnerable people safe from SARS-CoV-2 during the pandemic. Until further information, the focus of the dental health care providers should be on maximum application of safety regulations and recommendations by their local dental boards.

### Immunization

By far, three different types of coronaviruses (SARS-CoV, MERS-CoV, and SARS-CoV-2) have considerably affected global health in about 20 years; nonetheless, there is still no approved vaccines for these viruses [88]. Although currently numerous preclinical and clinical trials are being conducted with some promising results [88], it is noteworthy that one major drawback is that RNA viruses usually have higher mutation rates compared to DNA viruses, resulting in challenges for vaccine development [89]. Nevertheless, numerous pharmaceutical companies are actively involved in the different stages of vaccine development. In case of the development of an effective vaccine, dental health care providers, unarguably, should be among the first groups of professionals who receive the vaccine.

## Rapid point of care tests in dental offices

Currently, two major types of testing are available according to centers for disease control and prevention: viral testing and antibody testing indicating if the person does have a current infection or indicating if there was a previous infection, respectively. American Dental Association newsletter on April 17, 2020 urged dentists to be cautious about using novel coronavirus diagnostic tests before they have been properly evaluated and made available for dentists. Meanwhile, American Dental Association has sought federal recognition that licensed dentists may administer point of service tests authorized by the Food and Drug Administration (FDA) for novel coronavirus (SARS-CoV-2); however, because of the medical demand, currently, the medical suppliers are not planning on providing point-of-care tests to dentists in the very near future. It is noteworthy that at the present time, American Dental Association does not consider SARS-CoV-2 testing to be a scope of practice issue for dental offices according to ADA newsletter abovementioned. Although there is currently no FDA-approved or cleared test to diagnose or detect SARS-CoV-2, findings of some studies [90–93] may direct the future research into cheaper, faster, and more effective testing methods that would be available for the dental practices. A study conducted in a Hong Kong hospital reported consistent detection of coronavirus in the saliva of patients admitted from the first day that they were hospitalized [94]. In order to collect the samples, patients were instructed to cough out saliva from the throat into a sterile container that was later sent to the lab for analysis. This study underscored the advantage of simple and safe saliva sampling in a pandemic situation that can be actually utilized safely not only in the dental offices but also in anywhere including but not limited to busy clinics, airports, etc. [90]. It is of paramount importance that any testing method must strongly reduce the risk of SARS-CoV-2 transmission. Since Saliva may play a crucial role in the human-to-human transmission, salivary diagnostics might be an easy and cost-effective point-of-care platform for SARS-CoV-2 diagnosis because saliva self-collection will likely reduce the risk of SARS-CoV-2 transmission [91]. Additionally, the nasopharyngeal and oropharyngeal collection results in discomfort and possible bleeding especially in infected patients with thrombocytopenia that is potentially dangerous [91]. Our profession needs to emerge from this pandemic situation and probably enter a new world so maybe this testing method would become one of the routines in our daily practices.

## Conclusion

This article seeks to provide an overview of existing scientific evidence to suggest a guideline for reopening dental offices. We believe that studying this article and paying attention to its instructions can provide readers with an overview of the arrangements that should be considered for the gradual reopening of dental offices.

## Data Availability

The datasets generated and/or analyzed during the current study are not publicly available due but are available from the corresponding author on reasonable request.

## Abbreviations

ADA: American Dental Association
APF: Assigned Protection Factor
CDC: Centers for Disease Control and Prevention
CFR: Code of Federal Regulations
CBCT: Cone-beam computed tomography
CoVs: Coronaviruses
DHCP: Dental health care providers
DNA: Deoxyribonucleic acid
FCV: Feline calicivirus
FFRs: Filtering facepiece respirators
FFP: Filtering facepiece
FDA: Food and Drug Administration
HAI: Healthcare-associated infections
HCP: Health care providers
HC1: Hydrochloric acid
HPV: Hydrogen peroxide vapour
HOC1: Hypochlorous acid
IC: Infection control
ICP: Infection control practices
IUVA: International Ultraviolet Association
KPa: Kilo pascal
MPa: Mega pascal
MERS-CoV: Middle East respiratory syndrome coronavirus
MTA: Mineral trioxide aggregate
NIOSH: National Institute for Occupational Safety and Health
PAPR: Powered air-purifying respirator
RNA: Ribonucleic acid
ppm: Part per million
PPE: Personal protection equipment
RCT: Root canal therapy
SARS-CoV-2: Severe acute respiratory syndrome coronavirus 2
SARS-CoV: Severe acute respiratory syndrome-related coronavirus
VHP: Vaporized hydrogen peroxide
UV: Ultraviolet
